# A psychometric evaluation of the Functional assessment of cancer therapy—kidney symptom index (FKSI-19) among renal cell carcinoma patients suggesting an alternative two-factor structure

**DOI:** 10.1007/s11136-021-02839-9

**Published:** 2021-04-12

**Authors:** Andreas Karlsson Rosenblad, Pernilla Sundqvist, Bodil Westman, Börje Ljungberg

**Affiliations:** 1 Regional Cancer Centre Stockholm-Gotland, Region Stockholm, Box 6909, SE-102 39 Stockholm, Sweden; 2 Department of Medical Sciences, Division of Clinical Diabetology and Metabolism, Uppsala University, Uppsala, Sweden; 3 Department of Urology, Faculty of Medicine and Health, Örebro University, Örebro, Sweden; 4 Department of Care Science, Sophiahemmet University, Stockholm, Sweden; 5 Department of Surgical and Perioperative Sciences, Urology and Andrology, Umeå University, Umeå, Sweden

**Keywords:** Factor analysis, Health-related quality of life, Kidney cancer, Patient-reported outcomes, Psychometric analysis, Renal cancer carcinoma

## Abstract

**Purpose:**

To psychometrically evaluate the hypothesized four-factor structure of the 19-item Functional Assessment of Cancer Therapy—Kidney Symptom Index (FKSI-19) health-related quality of life (HRQoL) instrument in a sample of surgically treated renal cell carcinoma (RCC) patients and examine if an alternative factor structure with good psychometric properties may be derived from the available items.

**Methods:**

The model fit of the hypothesized four-factor structure was examined using confirmatory factor analysis on cohort data from 1731 individuals included in the National Swedish Kidney Cancer Register who had undergone surgery for RCC during the three years 2016–2018 and answered the FKSI-19 instrument within 6–12 months after surgery. Exploratory factor analysis was applied to the same dataset to derive a possible alternative factor solution.

**Results:**

The four-factor structure did not reach the thresholds for good model fit using the normed χ^2^-value or the Comparative Fit Index, although the Standardized Root Mean Square Residual and Root Mean Square Error of Approximation measures indicated good and acceptable model fits, respectively. An alternative 14-item trimmed FKSI version (FKSI-14) with a two-factor structure derived from the available FKSI-19 items was found to measure the same aspects of HRQoL as the full FKSI-19 instrument.

**Conclusion:**

The present study is the first to use psychometric methods for examining the factor structure of the FKSI-19 instrument. The hypothesized four-factor structure of FKSI-19 provided a barely acceptable model fit. The two-factor FKSI-14 structure may be used as an alternative or complement to the four-factor structure when interpreting the FKSI-19 instrument.

## Introduction

Kidney cancer has a yearly worldwide incidence of > 400 000 cases, corresponding to 2.2% of all cancers, with about 175 000 deaths occurring each year, equaling 1.8% of all cancer deaths [1]. Renal cell carcinoma (RCC) is the most common type of kidney cancer, accounting for > 90% of all cases [2]. The incidence of RCC is rising, in particular there is an increase in small renal tumors, mostly attributed to the increased use of tomographic imaging. Localized RCC at diagnosis is mainly treated with radical or partial nephrectomy and for smaller tumors also minimal invasive treatments as radiofrequency ablation [3]. During the last decade there has been a trend toward nephron sparing treatments as partial nephrectomy and ablative invasive treatments. The treatment of metastasized RCC at diagnosis is mainly based on systemic therapies including tyrosine kinase inhibitors and immunotherapies, but surgery of primary tumors and metastases should still be considered when immediate medical treatment is not required [4]. With estimated 5-year RCC survival rates ranging from 95% for stage I patients to 20% for stage IV patients [2], studying the health-related quality of life (HRQoL) experienced by the surviving patients is of large interest.

The general awareness and risk for recurrent disease will have an impact on the HRQoL for patients surgically treated for RCC. A number of treatment and surveillance factors will influence HRQoL. These include postoperative renal function, per- and postoperative complications, and positive surgical margins with increased risks for recurrent disease. Clinical management with adequate disease information and follow-up monitoring might be other factors of importance for the perception of well-being after RCC surgery.

Of the instruments available for measuring HRQoL among cancer patients, the Functional Assessment of Cancer Therapy—Kidney Symptom Index (FKSI) is the most kidney cancer-specific instrument, with the 19-item version (FKSI-19) being the most recent version [2, 5, 6]. FKSI-19 includes four subscales or domains/factors measuring different aspects of HRQoL among kidney cancer patients: Disease-Related Symptoms-Physical (DRS-P), Disease-Related Symptoms-Emotional (DRS-E), Treatment Side Effects (TSE), and Function/Well-Being (FWB). A comprehensive independent large-scale evaluation of the psychometric properties of the FKSI-19 instrument is, however, lacking. Specifically, psychometric methods have not previously been used for examining the factor structure of FKSI-19. The present study intends to rectify this shortcoming, using a large cohort of Swedish RCC patients.

### Aim

The aim of the present study was to evaluate the psychometric properties of the FKSI-19 instrument in a population of RCC patients treated with surgery, primarily by examining how well the observed data fitted the hypothesized four-factor structure of the FKSI-19 instrument, and secondarily by examining if an alternative factor structure with good psychometric properties may be derived from the available FKSI-19 items.

## Materials and Methods

### Study design and participants

The present study is based on data from the National Swedish Kidney Cancer Register (NSKCR) [7]. NSKCR has collected detailed data on diagnosis, tumor characteristics, and treatment of Swedish RCC patients since 2005, with the aim of measuring and improving the quality of care for RCC patients. While participation in NSKCR is voluntary, the coverage is 99% when compared to the national Swedish Cancer Register, to which reporting is mandatory [7, 8]. Details about inclusion and content of NSKCR have been published previously [8–11].

Data on FKSI-19 have been collected six months after surgery for all NSKCR participants undergoing surgical treatment for RCC since January 2016. The present study aimed at including all patients undergoing surgical treatment for RCC during the three years 2016-2018 who answered the FKSI-19 instrument within 6–12 months after surgery, i.e., until December 31, 2019. Surgical treatment was open, laparoscopic radical or partial nephrectomy performed by open laparoscopic or robot assisted laparoscopy or thermal ablation of the tumor.

**Fig. 1:**
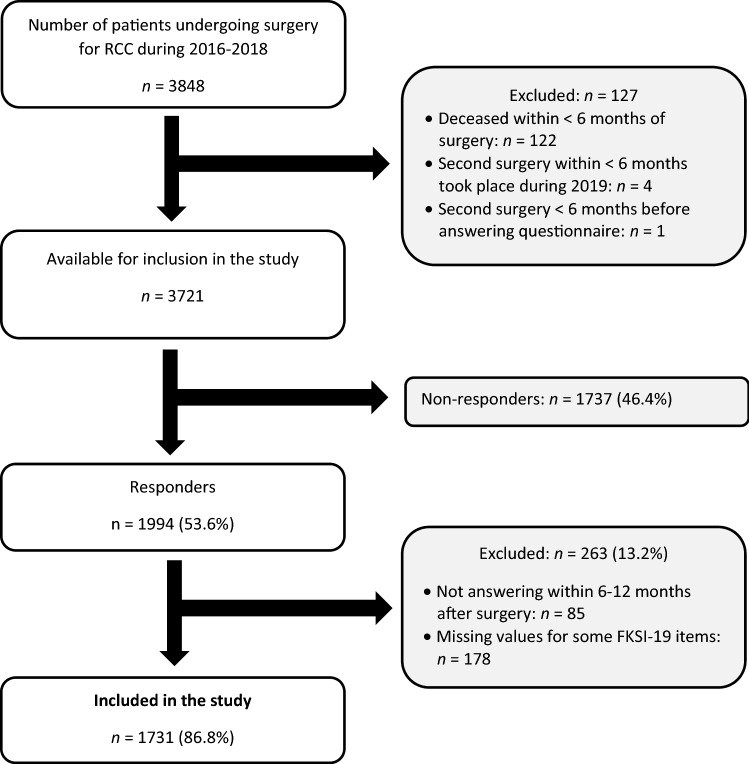
Flowchart of the inclusion process

Of the 3848 individuals who underwent surgery for RCC during the years 2016–2018, 127 individuals were excluded since they died within < 6 months of the surgery (*n* = 122), had a second surgery during 2019, within < 6 months of the prior surgery (*n* = 4), or had a second surgery < 6 months before answering the FKSI-19 instrument (*n* = 1). Thus, in total, 3721 individuals were available for inclusion in the study, of which 1994 (53.6%) answered the FKSI-19 questions. After excluding responders whose answers were not given within the time frame of 6-12 months after surgery (*n* = 85) or had missing values for some FKSI-19 items (*n* = 178), a total of 1731 individuals remained, thus constituting the study sample of the present study. A flow chart describing the inclusion process for the present study is given in Figure 1.

### Data collection

The invitation to participate in the study were sent out by regular mail together with an information letter. The information letter described the purpose of the study and included a personal code for answering a questionnaire containing the FKSI-19 instrument and a few study-specific questions via a secure internet link. It also explained that participation was voluntary and how confidentiality was ensured. One reminder, including a printed questionnaire and a pre-stamped envelope, was sent to those not responding within one month. Contact information to a research assistant was included, giving potential participants the possibility to ask questions about the questionnaire. The study-specific questions covered demographic characteristics such as marital status and highest level of education achieved (Primary school/Upper secondary school/College or university), in addition to questions about the patient’s contact with the health care system. Moreover, by linking the questionnaire to the NSKCR using each individual’s unique national Swedish Personal Identification Number (PIN) [12], information about sex, birthdate, date and type of surgery as well as date of death was obtained for each participant.

### FKSI-19

The FKSI-19 instrument [5, 6] consists of 19 items, with answers given on a five-level Likert-scale (“Not at all”, “A little bit”, “Somewhat”, “Quite a bit”, “Very much”), scored as either 0 to 4 (5 items) or reverse-scored as 4 to 0 (14 items). The resulting scores are summarized into a total score with a range of 0–76 points as well as separate domain scores for the four domains Disease-Related Symptoms-Physical (DRS-P; 12 items, score range 0-48 points), Disease-Related Symptoms-Emotional (DRS-E; 1 item, score range 0-4 points), Treatment Side Effects (TSE; 3 items, score range 0–12 points), and Function/Well-Being (FWB; 3 items, score range 0-12 points). A high score indicates less symptoms, i.e., a score of 0 indicates a severely symptomatic patient, while the highest possible score indicates a fully asymptomatic patient.

### Statistical analyses

Categorical data are presented as frequencies and percentages, *n* (%), while ordinal and continuous data are given as means and standard deviations (SDs). Marital status was categorized as married/cohabiting (yes/no) while type of surgery was categorized as open surgery (yes/no). Confirmatory factor analysis (CFA) was used to examine if the observed data fitted the hypothesized four-factor structure of the FKSI-19 instrument. To examine if other factor structures with better psychometric properties might be derived from the FKSI-19 items, an exploratory factor analysis (EFA) was applied to the dataset to elucidate its underlying factor structure.

For the CFA analyses, standardized factor loadings were used, and the overall model fit was assessed using a χ^2^ test, with a normed χ^2^ value < 5.0 considered an acceptable model fit. The goodness-of-fit indices Comparative Fit Index (CFI), Root Mean Square Error of Approximation (RMSEA), and Standardized Root Mean Square Residual (SRMR) were used as heuristic measures of model fit. Values of CFI > 0.90 and SRMR < 0.08 were considered indicating good model fits, while RMSEA < 0.05 and RMSEA < 0.08 were considered good and acceptable, respectively, model fits. R^2^ values were used to measure item reliability, with values of R^2^ ≥ 0.40 considered indicating an acceptable item reliability [13–15].

For the EFA analyses, the goal was to obtain a factor solution with easily interpretable factors and good psychometric properties, defined as all factors having ≥ 3 items with high factor loadings (absolute value > 0.4 after varimax rotation), with no item having high factor loadings for more than one factor. To start with, the number of factors to extract was determined using Velicer’s minimum average partial (MAP) criterion [16] and the EFA model was estimated using the minimum residual method, followed by varimax rotation. The items with lowest maximal absolute value factor loadings < 0.4 were then removed one at a time until a satisfactory factor solution according to the above-mentioned criteria was obtained.

All statistical analyses were performed using R 4.0.0 (R Foundation for Statistical Computing, Vienna, Austria), with CFA and EFA analyses performed using the R packages ‘lavaan’ version 0.6.7 and ‘psych’ version 2.0.7, respectively [17, 18]. For all statistical tests, two-sided *P* values < 0.05 were considered statistically significant.

## Results

Demographic and clinical symptoms characteristics for the 1731 patients with RCC that participated in the present study are given in Table 1, together with total and domain scores for the FKSI-19 instrument, overall and divided by sex. Almost two-thirds (*n* = 1133, 65.5%) of the patients were males, which was in line with the overall proportion of males (61.5%) in the NSKCR [8]. The participants were at a mean (SD) age of 66.7 (11.0) years at the time of surgery, with the questionnaire being answered a mean (SD) 8.0 (1.2) months after surgery. Most patients (72.7%) were married or cohabiting, while less than a third (29.8%) had a college or university education. Most patients were at TNM stages T1 (66.4%), N0 (90.1%), and M0 (93.5%), while open surgery was performed on almost half (49.1%) of the patients. The mean (SD) total score of 60.8 (10.6) points on FKSI-19 indicated an overall low degree of symptoms. The differences between males and females were in general small for all variables except marital status, where 77.4% of males were married/cohabiting, compared to 63.9% for females.

**Table 1:** Characteristics of the *n* = 1731 participating patients

		Overall	Men	Women	Missing
Characteristic	Variable	*n* = 1731	*n* = 1133	*n* = 598	*n* (%)
Demographic	Age at surgery (years), mean (SD)	66.7 (11.0)	66.7 (10.9)	66.7 (11.1)	0 (0.0)
	Time from surgery to answering questionnaire (months), mean (SD)	8.0 (1.2)	7.9 (1.2)	8.0 (1.2)	0 (0.0)
	Married/cohabiting, *n* (%)	1238 (72.7)	862 (77.4)	376 (63.9)	29 (1.7)
	Education level, *n* (%)				24 (1.4)
	Primary school	577 (33.8)	371 (33.2)	(4.9)	
	Upper secondary school	621 (36.4)	408 (36.5)	(6.1)	
	College or university	509 (29.8)	338 (30.3)	171 (29.0)	
Clinical	Open surgery, *n* (%)	847 (49.1)	566 (50.1)	281 (47.1)	5 (0.3)
	T stage, *n* (%)				4 (0.2)
	T1	1147 (66.4)	736 (65.1)	(9.0)	
	T2	202 (11.7)	137 (12.1)	(10.9)	
	T3	357 (20.7)	242 (21.4)	(9.3)	
	T4	(0.9)	111.0)	4(0.7)	
	TX	6 (0.3)	5 (0.4)	1 (0.2)	
	N stage, *n* (%)				0 (0.0)
	N0	1560 (90.1)	(89.5)	546(91.3)	
	N1	46 (2.7)	(2.6)	16(2.7)	
	N2	1 (0.1)	0 (0.0)	1 (0.2)	
	NX	124 (7.2)	89 (7.9)	35 (5.9)	
	M stage, *n* (%)				0(0.1)
	M0	1617 (93.5)	1061 (93.7)	556 (93.0)	
	M1	113 (6.5)	71 (6.3)	42 (7.0)	
FKSI-19	Disease-related symptoms-Physical (0–48 points), mean (SD)	38.8 (6.7)	39.2 (6.6)	38.2 (7.0)	0 (0.0)
	Disease-related symptoms-Emotional (0–4 points), mean (SD)	2.6 (1.2)	2.7 (1.2)	2.4 (1.2)	0 (0.0)
	Treatment side effects (0–12 points), mean (SD)	11.0 (1.7)	11.0 (1.7)	10.9 (1.8)	0 (0.0)
	Function/Well-Being (0–12 points), mean (SD)	8.4 (2.9)	8.4 (2.9)	8.4 (2.9)	0 (0.0)
	Total (0–76 points), mean (SD)	60.8 (10.6)	61.3 (10.3)	59.9 (10.9)	0 (0.0)

*SD* standard deviation

### Confirmatory factor analysis

A CFA analysis applying the hypothesized four-factor structure of the FKSI-19 instrument on the 1731 participants in the present study resulted in a χ^2^ value of 1550 with 147 degrees of freedom, giving a normed χ^2^ value of 10.5, thus not reaching the standard threshold for an overall good model fit. Neither did the CFI-value of 0.885 imply a good model fit. However, the SRMR-value of 0.055 did indicate a good model fit, while the RMSEA-value of 0.074 (90% CI 0.071–0.078) indicated an acceptable model fit. Testing the hypothesis that RMSEA ≤ 0.05 resulted in a *P* value < 0.001, clearly rejecting the hypothesis of a good model fit.

Factor loadings and item reliabilities for FKSI-19 are given in Table 2. For DRS-P, the strongest influence of the latent factor was on the indicators GP1 (“I have a lack of energy”), HI7 (“I feel fatigued”), and HI12 (“I feel weak all over”), with each 1 SD increase in DRS-P implying 1.026-, 0.999-, and 1.001-point increases in GP1, HI7, and HI12 scores, respectively (all *P* < 0.001). These indicators were also the only ones in the DRS-P domain having item reliability R^2^ ≥ 0.40, the threshold for an acceptable item reliability. In fact, all three indicators had R^2^ values > 0.7, with the highest value 0.780 observed for HI7. Notably, the two indicators RCC2 (“I have had blood in my urine”) and BRM3 (“I am bothered by fevers”) had very low item reliability, with R^2^ values of 0.012 and 0.093, respectively. For the single-indicator DRS-E factor, the model predicts a 1.226-point increase in GE6 (“I worry that my condition will get worse”) score for each 1 SD increase in DRS-E, with an item reliability of 1.0. For the TSE factor, all three indicators had R^2^ values < 0.4, indicating less than acceptable item reliabilities. For the FWB factor, finally, all three indicators had R^2^ values > 0.4, indicating acceptable item reliabilities, with values of 0.827 and 0.789 observed for GF3 (“I am able to enjoy life”) and GF7 (“I am content with the quality of my life right now”), respectively. For each 1 SD increase in FWB, the model predicts increases of 0.958 and 1.023 points for the indicators GF3 and GF7, respectively.

**Table 2:** Results for confirmatory factor analysis of the FKSI-19 instrument. Item reliabilities ≥ 0.40 are given in bold

Factor	Indicator	Standardized factor loadings			Standardized estimate	Item reliability (R^2^)
		Estimate	95% CI	*P* value		
DRS-P	GP1^a^	1.026	0.978–1.073	< 0.001	0.843	**0.710**
	GP4^a^	0.498	0.455–0.541	< 0.001	0.527	0.278
	C2^a^	0.316	0.278–0.354	< 0.001	0.390	0.152
	HI7^a^	0.999	0.957–1.042	< 0.001	0.883	**0.780**
	B1^a^	0.678	0.630–0.727	< 0.001	0.614	0.377
	BRM3^a^	0.118	0.099–0.136	< 0.001	0.306	0.093
	BP1^a^	0.425	0.382–0.467	< 0.001	0.463	0.214
	L2^a^	0.319	0.274–0.364	< 0.001	0.336	0.113
	HI12^a^	1.001	0.958–1.045	< 0.001	0.875	**0.766**
	RCC2^a^	0.048	0.027–0.070	< 0.001	0.110	0.012
	C6	0.357	0.308–0.405	< 0.001	0.350	0.123
	GF5	0.378	0.324–0.433	< 0.001	0.331	0.110
DRS-E	GE6^a^	1.226	1.185–1.267	< 0.001	1.000	**1.000**
TSE	GP2^a^	0.409	0.371–0.446	< 0.001	0.592	0.351
	C5^a^	0.365	0.321–0.409	< 0.001	0.455	0.207
	GP5^a^	0.467	0.420–0.515	< 0.001	0.524	0.275
FWB	GF1	0.747	0.699–0.795	< 0.001	0.673	**0.453**
	GF3	0.958	0.917–0.998	< 0.001	0.909	**0.827**
	GF7	1.023	0.978–1.067	< 0.001	0.888	**0.789**

*CI* confidence interval, *DRS-E* disease-related symptoms-emotional, *DRS-P* disease-related symptoms-physical, *FWB* function/well-being, *TSE* treatment side effects

^a^Reverse-scored

### Exploratory factor analysis

For the EFA analysis, the MAP criterion suggested a two-factor solution (minimum MAP = 0.016). After removing five items without high factor loadings (C2, BRM3, L2, RCC2, and C5) one at a time, a satisfactory 14-item solution was achieved, consisting of two factors with 9 and five items, respectively, with high factor loadings, and no item having high factor loadings for more than one factor. The resulting solution is given in Table 3. A possible interpretation of the nine-item factor is that it measures the presence of physical and mental symptoms (PMS) among the RCC surgery patients, while the five-item factor measures daily life function and well-being (DLFWB). Notably, the latter corresponds to the FKSI-19 FWB domain with the addition of the DRS-P items C6 (“I have a good appetite”) and GF5 (“I am sleeping well”). This 14-item trimmed FKSI version (FKSI-14) could be scored in the same way as the original FKSI-19 instrument, resulting in a total score with a range of 0–56 points and domain scores with ranges of 0-36 points for the PMS domain and 0–20 points for the DLFWB domain.

**Table 3:** Results of exploratory factor analysis (EFA) of the 19 items of the FKSI-19 instrument, giving a 14-item two-factor solution (FKSI-14). The largest factor loading for each item is given in bold

Item	FKSI-19 domain	Question	FKSI-14 domain	F1	F2
GP1^a^	DRS-P	I have a lack of energy	PMS	**0.716**	0.370
GP4^a^	DRS-P	I have pain	PMS	**0.565**	0.175
HI7^a^	DRS-P	I feel fatigued	PMS	**0.784**	0.328
B1^a^	DRS-P	I have been short of breath	PMS	**0.555**	0.226
BP1^a^	DRS-P	I have bone pain	PMS	**0.485**	0.132
HI12^a^	DRS-P	I feel weak all over	PMS	**0.812**	0.304
C6	DRS-P	I have a good appetite	DLFWB	0.139	**0.514**
GF5	DRS-P	I am sleeping well	DLFWB	0.147	**0.459**
GE6^a^	DRS-E	I worry that my condition will get worse	PMS	**0.469**	0.183
GP2^a^	TSE	I have nausea	PMS	**0.428**	0.231
GP5^a^	TSE	I am bothered by side effects of treatment	PMS	**0.436**	0.139
GF1	FWB	I am able to work (include work at home)	DLFWB	0.264	**0.641**
GF3	FWB	I am able to enjoy life	DLFWB	0.300	**0.836**
GF7	FWB	I am content with the quality of my life right now	DLFWB	0.360	**0.773**

*DLFWB* daily life function and well-being, *DRS-E* disease-related symptoms-emotional, *DRS-P* disease-related symptoms-physical, *FWB* function/well-being, *PMS* physical and mental symptoms, *TSE* treatment side effects. The following FKSI-19 items were excluded: C2, “I am losing weight”; BRM3, “I am bothered by fevers”; L2, “I have been coughing”; RCC2, “I have had blood in my urine”; C5, “I have diarrhea”

^a^Reverse-scored

## Discussion

The main finding of the present study was that the hypothesized four-factor structure of the FKSI-19 instrument provided a barely acceptable model fit when applied to data from the NSKCR, implying that an alternative factor structure may be contemplated. Moreover, for a factor structure to have good psychometric properties, it is generally recommended that each factor should include ≥ 3 items [14], which is not the case for the hypothesized four-factor structure of the FKSI-19 instrument. An alternative 14-item two-factor solution (FKSI-14) with good psychometric properties was thus derived from the available FKSI-19 items. Notably, the five items that were excluded from FKSI-14 (C2, BRM3, L2, RCC2, and C5) were those items describing explicit physical symptoms (weight loss, fever, coughing, blood in urine, and diarrhea). In contrast, items included in FKSI-14 described more vague symptoms and feelings (e.g., lack of energy, pain, fatigue, weakness, and nausea). Moreover, it should be noted that the five excluded items all had low item reliability, with R^2^ values between 0.012 and 0.207. This suggests that FKSI-14 may be a more robust measure of HRQoL among RCC patients. Finally, it should be noted that the four-factor FKSI-19 structure and the alternative two-factor FKSI-14 structure overall seem to measure the same aspects of HRQoL, with the PMS domain of FKSI-14 largely over-lapping with the DRS-P, DRS-E, and TSE domains of FKSI-19, and the DLFWB domain of FKSI-14 over-lapping with the FWB domain of FKSI-19.

The exclusion of items describing explicit physical symptoms while retaining items describing more vague symptoms and feelings is logical considering that the former are objective signs of a disease while the latter are subjective signs, which are better suited for being measured by HRQoL instruments. In general, it may still be important to measure explicit physical symptoms, in order to provide the clinicians working with the patients with sufficient information regarding individual patient's symptom burden, but these questions could then be asked separately and need not be included in the calculation of the HRQoL index.

However, it should be noted that the vast majority of RCC patients undergo surgery as their primary, and in many cases only, treatment. Surgery gives a relatively short postoperative convalescence, after which patients are living a normal daily life. Moreover, the explicit physical symptoms measured by the five excluded items are rare in this particular group of patients. They are more important after radiation therapy or chemotherapy, treatments that seldom are used for RCC patients. Additionally, in the NSKCR, these specific questions are answered by patients after their first line of treatment, with many patients never suffering from relapse. Actually, in a later stage of the disease with several lines of systemic treatment, there might even be a risk of underestimation of symptoms if the clinician relies on the patient’s answers to these specific questions for his or her assessment.

### Comparison with previous studies

There are only a few available studies of the psychometric properties of the FKSI-19 instrument. Before the FKSI-19 version was presented by Rao et al. [6], initial 15-item (FKSI-15) and 10-item (FKSI-10) versions were discussed by Cella et al. [19], with the FKSI-15 version including the same items as the latter FKSI-19 version except items HI12, GP2, C5, and GF7, while the FKSI-10 version in addition excluded items BRM3, L2, RCC2, GF5, and GF1 [5]. These early FKSI versions were presented as simple single-domain symptom indices, without undergoing any formal factor analyses to elucidate the underlying factor structures of the instruments. A latter study extracted a 9-item DRS-P version from the 15 items included in FKSI-15, primarily based on experts assessments, with some input from kidney cancer patients [20]. This 9-item DRS-P version included the same items as the 12-item DRS-P version included in FKSI-19, except items HI12, C6, and GF5 [5].

The FKSI-19 version of Rao et al. [6] was an attempt to reconcile FKSI-15 with another HRQoL instrument for kidney cancer patients, the Renal Cell Carcinoma Symptom Index (RCC-SI) [21]. It divided the FKSI-19 items into seven domains, seemingly using qualitative assessments: Pain, Fatigue, Cardiopulmonary symptoms, Bowel/bladder symptoms, Nutritional health, Psychosocial functioning, and Treatment side effects. Rothrock et al. [5], finally, used expert assessments to assign each item to one of the four domains DRS-P, DRS-E, TSE, and FWB of the current official FKSI-19 version.

Notably, the present study is thus the first to use psychometric methods for examining the factor structure of the FKSI-19 instrument. Moreover, while the present study included 1731 patients, previous studies included far fewer patients: 34 patients in a scale construction sample and another 141 patients in a validation sample in studies by Cella et al. [19, 20] and 50 patients in studies by Rao et al. [6] and Rothrock et al. [5]

### Strengths and limitations

Among the strengths of the present study was the large sample size of 1731 patients answering the FKSI-19 questions during the relatively short period of three year, far surpassing the sample sizes of previous studies of FKSI-19, which should contribute to making the results of the present study more robust. Another strength was the collection of data using the NSKCR, ensuring a high national coverage and generalizability of the results to RCC surgery patients nationwide. Among the limitations of the present study was the decision to only include patients living in Sweden, making it harder to generalize the results to other countries. Another limitation was that no repeated administration of FKSI-19 was performed, meaning that the test-retest reliability of FKSI-19 could not be evaluated.

## Conclusions

The present study is the first to use psychometric methods for examining the factor structure of the FKSI-19 instrument. The hypothesized four-factor structure of FKSI-19 provided a barely acceptable model fit when applied to data from the National Swedish Kidney Cancer Register. An alternative 14-item two-factor structure (FKSI-14) with good psychometric properties derived from the available FKSI-19 items may be used as an alternative or complement to the four-factor structure when interpreting the FKSI-19 instrument. The usefulness of this two-factor structure should be tested on other datasets.

## Data Availability

Data from the NSKCR are available for researchers.

## Abbreviations

CFA: Confirmatory factor analysis
CFI: Comparative fit index
CI: Confidence interval
DLFWB: Daily life function and well-being
DRS-E: Disease-related symptoms-emotional
DRS-P: Disease-related symptoms-physical
EFA: Exploratory factor analysis
FKSI: Functional assessment of cancer therapy – kidney symptom index
FWB: Function/well-being
HRQoL: Health-related quality of life
MAP: Minimum average partial
NSKCR: National Swedish kidney cancer register
PIN: Personal identification number
PMS: Physical and mental symptoms
RCC: Renal cell carcinoma
RMSEA: Root mean square error of approximation
SD: Standard deviation
SRMR: Standardized root mean square residual
TSE: Treatment side effects
